# Challenges in Cancer Biomarker Discovery Exemplified by the Identification of Diagnostic MicroRNAs in Prostate Tissues

**DOI:** 10.1155/2020/9086829

**Published:** 2020-05-05

**Authors:** Filip Ambrozkiewicz, Jakub Karczmarski, Maria Kulecka, Agnieszka Paziewska, Magdalena Cybulska, Michal Szymanski, Jakub Dobruch, Artur Antoniewicz, Michal Mikula, Jerzy Ostrowski

**Affiliations:** ^1^Department of Genetics, Maria Sklodowska-Curie National Research Institute of Oncology, Warsaw, Poland; ^2^Department of Gastroenterology, Hepatology and Clinical Oncology, Centre of Postgraduate Medical Education, Warsaw, Poland; ^3^Uro-oncology Department, Maria Sklodowska-Curie National Research Institute of Oncology, Warsaw, Poland; ^4^Department of Urology, Centre of Postgraduate Medical Education, Independent Public Hospital of Professor W. Orlowski, Warsaw, Poland; ^5^Department of Urology, Multidisciplinary Hospital Warsaw-Miedzylesie, Warsaw, Poland

## Abstract

Identification and clinical translation of routinely tested biomarkers require a complex and multistep workflow. Here, we described a confirmatory process estimating the utility of previously identified candidate tissue miRNAs for diagnosis of prostate cancer (PCa). RNA was isolated from formalin-fixed paraffin-embedded (FFPE) prostate tissue surgically resected from 44 patients with PCa and 24 patients with benign prostate hyperplasia (BPH). Of the 92 RNA samples obtained, 68 represented 42 malignant (PCa) areas and 26 represented nonmalignant (PCa 0%) areas of the prostate tissue sections. The levels of miR-32-5p, miR-183-5p, miR-141-5p, miR-187-3p, miR-375, miR-663b, miR-615-3p, miR-205-5p, miR-221-3p, and miR-222-3p were evaluated using Exiqon chemistry. Five (miR-32-5p, miR-141-5p, miR-187-3p, miR-375, and miR-615-3p), one (miR-32-5p), and two (miR-32-5p and miR-141-5p) miRNAs discriminated between BPH and areas of cancer-bearing prostate tissue harboring different numbers of cancer cells (PCa 15–70%, PCa 2–10%, and PCA 0%, respectively), with an area under the receiver operating characteristics curve (AUC-ROC) > 0.9. Only miRNA 32-5p discriminated BPH specimens from sections of cancer-bearing prostate tissue with a low percentage, a high percentage, or no dysplastic cells. miR-32-5p could be considered as potential diagnostic biomarker discriminating BPH from noncancerous areas within cancer-bearing prostate tissue. However, further clinical studies are warranted to confirm its diagnostic utility.

## 1. Introduction

Identification and clinical translation of routinely tested biomarkers remain the challenge of modern medicine. Due to ease and reproducibility of extraction from biological samples, their stability and measurement accuracy by standard techniques, miRNA have been considered as valuable biomarker candidates. However, more than 50 miRNAs have been described to be involved in the development of prostate cancer (PCa) but their aberrant expression may fluctuate due to the molecular heterogeneity of prostate cancer [1].

Histological evaluation still remains the gold standard for cancer diagnosis. However, the ability to detect cancer, even when neoplastic cells are missed on needle biopsies, is a highly desirable attribute for a PCa biomarker. In cases of suspected PCa in which the initial biopsy is negative, such biomarkers may facilitate the clinical choice between watchful waiting and collection of additional biopsy samples.

A clinically and economically feasible biomarker requires an appropriate type of specimen, such as a formalin-fixed, paraffin-embedded (FFPE) tissue sample. Using frozen tissues, we described recently a diagnostic workflow for selecting miRNA biomarker diagnostic for PCa [2]. Deep sequencing of small RNA transcriptomes isolated from prostate specimens identified 123 miRNAs significantly dysregulated in PCa, of which 31 were dysregulated regardless of the dysplastic cell content of the specimen studied. Eight selected miRNAs were tested further by standard quantitative reverse transcription PCR (qRT-PCR) and Exiqon assays of the same sample sets. Of these, six (miR-9-3p, miR-9-5p, miR-187-3p, miR-32-5p, miR-183-5p, and miR-141-5p) miRNAs showed a moderate to high ability to discriminate between benign prostate hypertrophy (BPH) and noncancerous areas within cancer-bearing prostate tissue, with an area under the receiver operating characteristics curve (AUC-ROC) ranging from 0.829 to 1. However, validation experiments on frozen core biopsies confirmed differential expression of four miRNAs (miR-187-3p, miR-183-5p, miR-32-5p, and miR-141-5p) between BPH and PCa, with AUC-ROC values ranging only from 0.720 to 0.800.

Here, we used a set of independent FFPE tissues from Polish patients to assess the diagnostic value of the candidate biomarker miRNAs previously identified by us [2] and by Kristensen et al. [3].

## 2. Materials and Methods

The study examined 68 FFPE prostate tissues surgically resected at the Department of Urology at the Maria Sklodowska-Curie Institute-Oncology Centre, Medical Center for Postgraduate Education and Multidisciplinary Hospital Warsaw-Miedzylesie, Warsaw, Poland. None of the patients had received previous hormonal therapy or radiotherapy to the prostate. The study was performed in accordance with the ethical standards of the local bioethical committee and in accordance with the principles of the 1964 Declaration of Helsinki.

Prior to RNA extraction, several sections were prepared from different areas of all malignant and nonmalignant prostate tissues; the upper and lower sections from each set of sections were evaluated by referral pathologists to control for the relative cell type content. Of the 92 RNA samples obtained, 68 represented 42 malignant (PCa) and 26 represented nonmalignant (PCa 0%) prostate areas in tissue samples from 44 PCa patients (median age, 63 years; range, 51–76 years) and 24 BPH patients (median age, 68.5 years; range, 58–86 years).

RNA was isolated using a QIAamp RNA FFPE Tissue Kit (Qiagen), according to the manufacturer's protocol. The samples were submitted to QIAGEN Genomic Services, where each RNA sample was reverse transcribed (in triplicate) into cDNA and run on the miRCURY LNA miRNA PCR Custom panel. The levels of the following miRNAs were determined: (1) four miRNAs (miR-32-5p, miR-183-5p, miR-141-5p, and miR-187-3p) selected from our previous study [2]; (2) six miRNAs (miR-375, miR-663b, miR-615-3p, miR-205-5p, miR-221-3p, and miR-222-3p) selected from a 13-miRNA diagnostic classifier for PCa developed and validated by Kristensen et al. [3]; and (3) four miRNAs (miR-191-5p, miR-151a-5p, hsa-miR-423-3p, and hsa-miR-425-5p) selected for data normalization. Each individual amplification product in the PCR panel was scrutinized by melting curve analysis, calculation of the amplification efficiency, and comparison of the Cq value with the background level in the negative control sample.

## 3. Results

To select diagnostic miRNAs that could distinguish between BPH and cancerous prostatic tissue, the expression of previously identified candidate biomarkers [2] [3] was tested by a standard quantitative reverse-transcription PCR (qRT-PCR) using Exiqon chemistry. RNA was extracted from 24 BPH samples and from 68 specimens obtained from 44 prostates with malignant pathology. As ascertained by histological examination, 12 cancerous samples contained <10% (PCa < 10%) dysplastic cells and 30 contained >10% (PCa > 10%) dysplastic cells. Twenty-six samples obtained from adjacent noncancerous regions did not contain any dysplastic cells (PCa 0%).

Measurable qRT-PCR signals were obtained in all samples for ten candidate miRNAs and four miRNAs selected for data normalization. Since three miRNAs (miR-423-3p, miR-425-5p, and miR-191-5p) selected for the normalization were differentially expressed among samples, the data were normalized only in relation to the level of miR-151a-5p.

Ten, eight, and six of the studied miRNAs were differentially expressed (adjusted *P* value < 0.05) between BPH and PCa > 10% (2–10%), PCA < 10% (15–70%), and PCA 0% samples, respectively (Table 1). Of these, five, one, and two miRNAs exhibited a high ability to discriminate between BPH specimens and different parts of cancer-bearing prostates (PCa > 10%, PCA < 10%, and PCA 0%, respectively), with an area under the receiver operating characteristics curve (AUC-ROC) > 0.9 (range, 0.906–1) (Table 1). However, only miRNA 32-5p discriminated BPH specimens from both cancerous (with a low or high content of dysplastic cells) and noncancerous (PCa 0%) areas of cancer-bearing prostate tissue with an AUC-ROC close to 1.0 and, therefore, could be considered as a single tissue diagnostic biomarker for PCa.

## 4. Discussion

Selection of new biomarkers includes a discovery step, development of an assay, preliminary confirmation of clinical utility, and, finally, a clinical trial to assess the actual impact of the biomarker [4]. While an ideal biomarker should be binary and consistently and completely discriminate between diseased and normal tissue, most candidate biomarkers generate numeric values on a continuous scale, resulting in overlap between disease and normal states. Furthermore, initial results for biomarkers are often not reproduced in later studies due to issues with study design, assay platforms, and availability of specimens for biomarker evaluation [5, 6]. Thus, of the thousands of new potential diagnostic biomarkers reported every year, few add value to conventional cancer cytological and histological diagnostics [6].

A clinically and economically feasible biomarker requires an appropriate type of specimen, such as a formalin-fixed, paraffin-embedded (FFPE) tissue sample. Here, we reevaluated the diagnostic potential of four miRNAs selected by us with the use of massive sequencing of small RNAs isolated from frozen prostate tissues [2] and six miRNAs which were reported by others as discriminative in three PCa patient cohorts [3]. The ability to analyze FFPE tissue samples greatly increases the general applicability of biomarker assays [7]; therefore, in this study, we isolated RNA from FFPE prostate tissue samples. To note, while the small RNA fraction isolated from FFPE tissues may be contaminated by degraded mRNA fragments which hinders miRNA profiling by mass sequencing, it is sufficient for the analyses of individual miRNAs with the use of PCR-based techniques.

Accurate detection and quantification of miRNA depend on the technology platform used for testing [2]; therefore, to minimize technical variability, all tests were performed at a central laboratory employing reproducible and standardized technology. In addition to the statistical analysis of results undertaken as an integral part of assay documentation, multiple statistical tests were added by our own biostatisticians.

The present study highlights a discrepancy between the results from the initial miRNA biomarker discovery studies [2, 3] and the results obtained herein. Of the ten miRNAs evaluated, only miR-32-5p generated near-binary results with an AUC close to 1, regardless of whether the dysplastic cell content of tissue sections was high (between 15% and 70%), low (between 2% and 10%), or zero. In line with our findings, other studies suggest that the estimated success rate for development of an ideal biomarker that shows sufficient discriminatory power for an accurate diagnosis is as low as 0.1% [5, 6].

As reported previously, we found that increased expression of miR-32-5p is associated with tumorigenesis; indeed, miR-32 promotes proliferation and migration of both breast and PCa cells [8, 9]. Treatment of colorectal adenocarcinoma cells with fatty acids, and photodynamic therapy of oral cancer cells, downregulates expression of miR-32-5p [10, 11], and miR-32-5p is a specific mediator of bladder tumor survival [12]. In addition, miR-32-5p contributes to migration and invasion of colorectal carcinoma and hepatocellular carcinoma cells via downregulation of antioncogene phosphatase and tensin homologue (PTEN) [13, 14] and is involved in migration, invasion, and metastasis of pancreatic cancer cells [8]. Altered signaling via miR-32-5p/testicular nuclear receptor 4 (TR4) may promote metastasis of clear cell renal cell carcinoma [15]. Finally, miR-32-5p promotes gastric carcinoma tumorigenesis, modulates chemoresponsiveness of hepatocellular carcinoma to platinum-based agents by inducing multidrug resistance, and contributes to castration resistance, radioresistance, and chemoresistance of PCa via the miR-32-5p-Kruppel-like factor 4 (KLF4) signaling axis [16–19].

A suspicion of PCa results from elevated levels of serum prostate-specific antigen (PSA) and/or palpable alterations within the prostate upon digital rectal examination. Transrectal ultrasound- (TRUS-) guided prostate core biopsy is a standard procedure for PCa diagnosis. However, PCa is a multifocal disease, and even a 20-core biopsy approach can miss up to 10% of cancers [20]. In addition, PCa can mimic benign prostate glands [21]. Thus, more than 60% of biopsies conducted in response to a positive PSA test are negative [22]. Thus, miR-32-5p expression might be used to complement microscopic examination and clinical parameters. Its most important attribute would be its ability to identify PCa even when neoplastic cells are missed by needle biopsies or in cases of minimal residual cancer after radical prostatectomy. In other words, miR-32-5p may aid clinicians when deciding between watchful waiting and additional biopsy in cases of suspected PCa in which the initial biopsy is negative. However, this statement should be proved by a clinical trial which, unfortunately, is much more expensive and organizationally difficult. That is why our study lacks such a final assessment, necessary in the process of identifying the diagnostic biomarker of PCa.

## 5. Conclusions

While several miRNAs might distinguish cancerous from benign prostate tissue, miR-32-5p seems to be the best PCa biomarker (at least in Polish patients) as it can discriminate benign prostate tissue from noncancerous areas within cancer-bearing prostates. However, before a miR-32-5p-based biomarker will be ready for clinical use, further studies on its clinical benefits (including comparative- and cost-effectiveness) are required. In conclusion, this study confirms that miRNA is a stable biomarker for PCa, even in FFPE tissue samples, which are a suitable source of material for miRNA diagnostic assays. However, the dependency of miR-32-5p on tissue fixation methods and sample age (both of which affect miRNA integrity) should be evaluated.

## Figures and Tables

**Table 1:** Differentially expressed miRNAs in the PCa and BPH groups.

miRNA name	PCa > 10%*vs.* BPH			PCa < 10%*vs.* BPH			PCa 0% *vs.* BPH		
	*P* adjusted	FC	AUC	*P* adjusted	FC	AUC	*P* adjusted	FC	AUC
hsa-miR-32-5p	1.94*E*-26	0.57	**1.000**	5.17*E*-15	0.72	**0.983**	2.23*E*-24	0.66	**0.996**
hsa-miR-141-5p	2.11*E*-25	0.73	**0.985**	1.44*E*-08	0.83	0.848	1.36*E*-16	0.83	**0.901**
hsa-miR-187-3p	1.56*E*-18	1.64	**0.906**	7.33*E*-09	1.34	0.859	4.83*E*-08	1.27	0.767
hsa-miR-183-5p	2.54*E*-13	0.81	0.839	0.595608	1.02	0.468	0.004219	1.08	0.640
hsa-miR-375	1.43*E*-23	2.41	**0.964**	0.000134	1.50	0.737	0.013417	1.24	0.631
hsa-miR-615-3p	1.04*E*-12	0.72	**0.963**	2.76*E*-07	0.82	0.884	0.000463	0.89	0.757
hsa-miR-222-3p	3.45*E*-14	0.41	0.848	0.000164	0.77	0.728	0.716311	0.98	0.514
hsa-miR-221-3p	2.28*E*-13	0.51	0.835	0.001312	0.82	0.691	0.377882	0.95	0.541
hsa-miR-205-5p	2.02*E*-12	0.76	0.822	0.131802	0.95	0.592	0.747734	0.98	0.478
hsa-miR-663b	2.43*E*-08	0.64	0.751	0.046259	0.88	0.617	0.17529	0.92	0.565

FC: fold change in gene expression; AUC: area under the curve. In bold are the AUC values greater than 0.9.

## Data Availability

The qPCR dataset generated during the current study are available from the corresponding author on reasonable request.
